# Contributing Factors in Whether Displaced Households Want to Receive Humanitarian Information from Humanitarian Actors: Iraq Multi-Cluster Needs Assessment

**DOI:** 10.3390/ijerph191610114

**Published:** 2022-08-16

**Authors:** Jin-Won Noh, Jooyoung Cheon, Kyoung-Beom Kim, Si Eun Song, Jiho Cha, Young Dae Kwon

**Affiliations:** 1 Division of Health Administration, College of Software and Digital Healthcare Convergence, Yonsei University, Wonju 26493, Korea; 2 Department of Nursing Science, Sungshin Women’s University, Seoul 02844, Korea; 3 Department of Health University, Dankook University, Cheonan 31116, Korea; 4 Industry-University Cooperation Foundation, Soonchunhyang University, Asan 31538, Korea; 5 Moon Soul Graduate School of Future Strategy, Korea Advanced Institute of Science and Technology, Daejeon 34141, Korea; 6 Department of Humanities and Social Medicine, College of Medicine and Catholic Institute for Healthcare Management, The Catholic University of Korea, Seoul 06591, Korea

**Keywords:** Coronavirus disease 2019 (COVID-19), internally displaced persons, returnees, humanitarian information, humanitarian assistance

## Abstract

Due to political conflict, insurgency, and the COVID-19, the number of displaced households in need of humanitarian support in Iraq has increased. This study investigated factors related to desire of displaced households to receive humanitarian information. Data from the eighth round of the Iraq Multi-Cluster Needs Assessment was used. We classified the household displacement status, identifying levels and types of humanitarian information that the households sought, together with whether the households were impacted by COVID-19. We identified safety and security, housing, water and electricity services, education, health care, and levels of humanitarian assistance resulted in significant differences between internally displaced person (IDP) and returnee households in terms of interest in receiving humanitarian information. The desire to receive humanitarian information was related to whether household members were unemployed due to COVID-19, displacement status, and walking time to reach the nearest health care facility and marketplace. Returnees and IDPs in Iraq are facing a new crisis. Their individual, structural, and environmental vulnerabilities are increasing commensurately. New strategies such as strategies using online or mobile communication that provide humanitarian information are needed to provide humanitarian information to vulnerable groups such as those who have lost jobs due to COVID-19, female heads of households, and those with health problems. In addition to traditional cash and voucher support, the use of the latest technologies such as smartphones and mobile clinics in humanitarian settings would be new strategies.

## 1. Introduction

As COVID-19 continues to spread around the world, various aspects of our daily lives have been seriously disrupted. In particular, the Middle East, which has long experienced ongoing conflict and wars, has been severely damaged by this pandemic. The combined consequences of war, conflict, and crisis in public health are too burdensome for the country to overcome [1,2,3]. Iraq, which suffers uniquely from recurring wars and conflicts, has been affected by the pandemic just like every other region of the world [3,4,5,6]. Due to the Iraq conflict and COVID-19 pandemic, the number of displaced households in need of national humanitarian support has increased [4,5,7]. According to previous studies, forcibly displaced people have been economically disadvantaged since before the COVID-19 pandemic, but they may have been seriously affected in terms of economics due to measures to prevent the spread of the virus [8]. It has become more difficult for refugees to access the labor market, social safety nets, and supports provided by humanitarian organizations due to COVID-19 pandemic, further worsening the economic situation of forcibly displaced people, thereby exacerbating their livelihood difficulties and poverty.

According to a recent report, about 2.4 million people in Iraq are in acute need, including internally displaced persons (IDPs) and returnees. Two-thirds of people in need have described their humanitarian conditions as severe, extreme, and catastrophic [3]. Infectious disease outbreaks exacerbate these humanitarian conditions. The proportion of IDPs and returnees has increased due to loss of employment and relatively high prices of food [3]. In many countries, including Iraq, mental health, women’s health, and communicable diseases are the main health needs of refugees. Specifically, many refugees report suffering from health problems, such as injuries, posttraumatic stress disorder (PTSD), depression, anxiety, hypertension, diabetes, cardiovascular diseases, respiratory diseases, gynecological problems, communicable diseases, or musculoskeletal conditions [2,3,9,10,11]. Nevertheless, most refugees have barriers to accessing health care, including geographic barriers, lack of awareness, and lack of education [9,12].

Humanitarian agencies can establish and provide support in various areas, such as health care, camp management, or shelter for displaced households. Unfortunately, most humanitarian agencies have been adversely impacted by the COVID-19 pandemic and have had to curtail their activities due to reduced funding [1,2,3]. Challenges to humanitarian aid could remain ongoing for the foreseeable future. To adequately provide limited humanitarian resources to the places and people most in need, it is important to identify levels of information and assistance needed according to specific conditions of displaced households.

Even though the need for information on the most urgent and important humanitarian aid due to the pandemic situation and limited resources, there is little research on this [12,13]. Since the majority of IDPs do not live in camps, research on them is not easy, so there are few studies, particularly regarding the health of IDPs [12]. Few studies have been conducted on the characteristics of displaced people who want to receive humanitarian information. Identifying vulnerable groups helps develop new strategies to deliver humanitarian information to displaced populations as efficiently as possible within the constraints of limited resources. Accordingly, the present study investigates what types of information are reported as being top priority among displaced households and determines which factors are related to whether or not displaced households want to receive humanitarian information from humanitarian actors.

## 2. Materials and Methods

### 2.1. Design and Data Collection

The REACH Initiative, which endeavours to provide data and analysis from contexts of crisis to inform humanitarian strategies, conducted the eighth round of the Iraq Multi-Cluster Needs Assessment (MCNA) to identify multiple sectors of need in conflict-affected populations across Iraq, together with the Assessment Working Group (AWG), United Nations Office for the Coordination of Humanitarian Affairs (OCHA), and the Inter-Cluster Coordination Group (ICCG). The MCNA VIII conducted phone-based household surveys in consideration of governmental restrictions on movement and access due to COVID-19. Interviewees were individual representatives of whole households. The MCNA VIII data were based on a non-probabilistic approach driven by quota-based sampling. The survey was conducted from 14 July to 23 September 2020. A total of 3872 households participated, comprising households represented by IDPs and returnees. After excluding 198 households with incomplete information from household heads, 3674 households were included in the analysis.

### 2.2. Variables

The variables contained the demographic characteristics of displaced households (number of family members) and specific vulnerabilities of each head of household (HOH), including features such as being elderly, being female, and/or being an HOH with health problems which were contributing factors to vulnerabilities that were reported in prior literature [14]. Employment status (working status of HOH) and household total income were included as socioeconomic factors. We identified household displacement status. We asked what kind of humanitarian information each household would like to receive from humanitarian actors. We also asked households whether they had had insufficient food anytime in the last 30 days. To assess the impact of COVID-19, we asked households whether they had experienced movement restrictions due to COVID-19 and whether any household members had lost their jobs because of the COVID-19 pandemic. To evaluate accessibility to health care facilities, the walking time of respondents to nearest health care facilities was investigated. We also investigated respondents’ walking time to nearest marketplaces to evaluate their accessibility.

### 2.3. Ethical Consideration

This study was conducted with ethics approval by the Institutional Review Board of Dankook University (IRB No. DKU-IRB-NON2020-006) in the category of human-subject database research.

### 2.4. Statistical Analysis

Descriptive analyses were conducted to summarize the humanitarian needs, COVID-19 impacts, vulnerabilities, and demographic characteristics of Iraqi displaced households. We performed inter-population subgroup comparisons using Rao-Scott corrected chi-squared analyses for categorical data and design-weighted univariable linear regression for numerical data. Results are presented as sample frequencies and weighted proportion estimates with 95% confidence intervals (CIs) where appropriate. Multivariable binary logistic regression analysis was performed to explore the factors associated with humanitarian needs among Iraqi displaced households. Adjusted odds ratios (ORs) with 95% CI estimates were reported. Statistical software Stata/MP version 16.1 (Stata Corp LP, College Station, TX, USA) was used for data processing and statistical analyses.

## 3. Results

Among a total of 3674 representative samples of displaced households in Iraq, 1513 were returnee households originating from regions other than those where the interviews were conducted. The type of humanitarian information most desired by respondents was options for livelihood. It was identified that desires for safety and security, status of housing, water services, electricity services, education, health care, and levels of humanitarian assistance were significantly different between IDP and returnee households (*p <* 0.001). Internally displaced person households indicated a desire for information about livelihoods (64.8%), humanitarian support (63.7%), and health care (40.4%). In comparison, returnee households indicated interest in humanitarian information about livelihoods (68.2%), status of housing (36.6%), and safety and security (35.5%). In terms of governmental restrictions on movement due to COVID-19, requirements to show identity documents (*p =* 0.002) and requirements to provide a specific reason for movement (*p <* 0.001) differed significantly between IDP and returnee households. Also of note, 40.2% of IDP households and 10.4% of returnee households experienced job loss among household members due to COVID-19, indicating a statistically significant difference between the groups (*p <* 0.001).

Related to heads of IDP households, 8.0% had an elderly-headed household and 12.4% had a female-headed household. Again, in IDP households, 34.9% reported household heads with health problems (injury, chronic/communicable disease, and other examples). At 68.3%, the majority of IDP HOHs reported being employed (working), and the IDP household mean total monthly income was 455,000 Iraqi dinar (IQD). Walking time to reach the nearest health care facility was less than 15 min for 59.5% of IDP households. As for walking time to reach the nearest marketplace, 83.4% of IDP households were within 15 min. Regarding heads of returnee households, 5.0% were elderly-headed and 9.7% were female-headed. In returnee households, 34.9% reported heads of household with health problems. In terms of employment status and income levels, 81.4% of returnee HOHs reported being employed (working), with a mean total income of 495,000 IQD. Regarding walking time to reach the nearest health care facility, 54.8% of returnee households were within 15 min. As for walking time to reach the nearest marketplace, 75.9% of returnee households reported being within 15 min. In identifying household demographic differences and vulnerability covariate differences between IDP households and returnee households, all factors of mean number of family members (*p <* 0.001), elderly-headed households (*p <* 0.05), HOH working status (*p <* 0.001), walking time to reach the nearest health care facility (*p <* 0.001), and walking time to reach the nearest marketplace (*p <* 0.005) were significantly different between the groups (Table 1).

**Figure 1:**
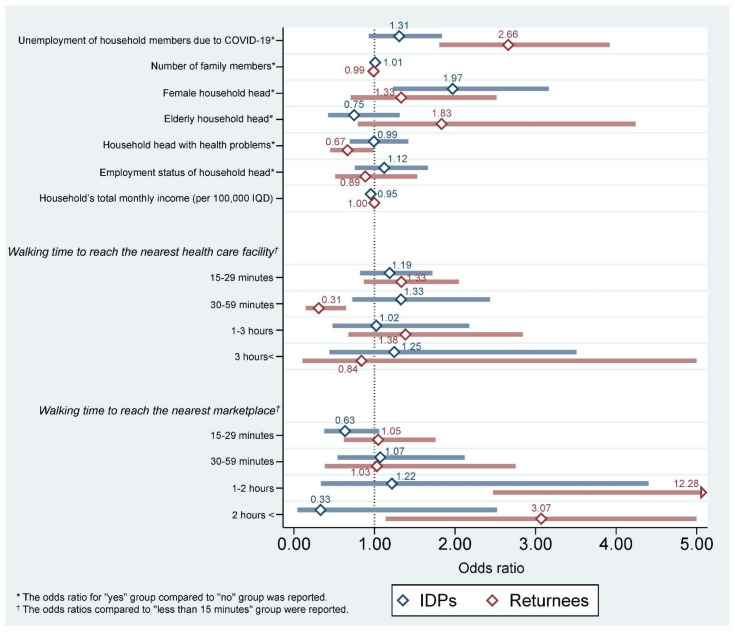
Factors associated with humanitarian information needs among Iraqi displaced households.

Table 2 summarizes factors associated with humanitarian information needs among Iraqi displaced households. Households with members who had lost jobs due to COVID-19 (total: OR = 2.061, *p* < 0.001, 95% CI = 1.569 to 2.708; returnees: OR = 2.661, *p <* 0.001, 95% CI = 1.805 to 3.922) and IDP households (returnees: OR = 0.325, *p* < 0.001, 95% CI = 0.255 to 0.414) showed significantly higher needs for humanitarian information. Female HOHs (OR = 1.971, *p* = 0.005, 95% CI = 1.228 to 3.164) and households with lower total monthly income (OR = 0.953, *p* = 0.008, 95% CI = 0.920 to 0.987) were significantly associated with a higher need for humanitarian information only among IDP households. Heads of household with health problems (OR = 0.666, *p* = 0.044, 95% CI = 0.449 to 0.988) were significantly associated with a lower need for humanitarian information only among returnee households. Households that were able to reach the nearest health care facility in 30 to 59 min by walking had lower humanitarian information needs than households that were able to reach the nearest health care facility in less than 15 min (total: OR = 0.454, *p* = 0.003, 95% CI = 0.272 to 0.759; returnees: OR = 0.308, *p* = 0.002, 95% CI = 0.146 to 0.649). Households that were able to reach the nearest marketplace in more than 15 min by walking had significantly higher humanitarian information needs than households that were able to walk to the nearest marketplace in less than 15 min (internally displaced persons/one to two hours: OR = 11.569, *p* = 0.003, 95% CI = 2.358 to 56.769; internally displaced persons/more than two hours: OR = 2.789, *p* = 0.023, 95% CI = 1.153 to 6.747; returnees/one to two hours: OR = 17.106, *p* = 0.004, 95% CI = 2.476 to 120.500; returnees/more than two hours: OR = 3.070, *p* = 0.027, 95% CI = 1.138 to 8.281) (Table 2) (Figure 1).

## 4. Discussion

Due to political conflict and insurgency and the COVID-19 pandemic, the number of displaced households in need of national humanitarian support has increased in Iraq [5,6,7]. Because humanitarian resources are limited, however, it is important to identify types of information and levels of assistance needed based on the specific conditions of displaced households. Accordingly, the present study performs multivariate binomial logistic regression analysis with MCNA VIII data to investigate factors related to desire for humanitarian information from humanitarian actors in displaced households.

Our findings highlight that information about livelihoods was the top priority in both IDP and returnee households. The next most important types of information were humanitarian support and health care for IDPs and housing status and safety/security for returnees. These results are similar to those of a previous study conducted on refugees from six African countries [15]. According to the study, refugees were struggling with economic difficulties, limited livelihoods, lack of access to services, and health-related and security risks due to COVID-19 pandemic. IDPs receive more protection than returnees from the government because IDPs remain in their home countries and become refugees. Nevertheless, IDPs often move to areas where humanitarian aid is difficult, making them a vulnerable group [3,16]. Returnees have been subject to strict restrictions on movement by the government in the context of the COVID-19 pandemic. They report needing humanitarian aid in more basic and general areas such as housing, safety, electricity services, and water supply. High levels of unemployment and the high price of food have increased the proportion of IDPs and returnees with acute needs [3]. Regardless, humanitarian aid is likely to remain limited for a while insofar as most humanitarian agencies have decreased their activities due to reduced funding caused by the COVID-19 pandemic [1,2,3].

In this study, unemployment in returnee households due to COVID-19 is shown to be related to increased demand for humanitarian information in Iraq. The fragile and weakened socioeconomic state has led to widespread unemployment and food insecurity, causing a higher demand for humanitarian aid in displaced households than in the past [3,4,5,6]. Refugees report that they have had difficulties finding jobs due to discrimination and other physical/financial barriers [3,10]. Previous research has shown that forcibly displaced people who have been economically affected by the COVID-19 pandemic coped with their income loss by reducing their food consumption [8]. Refugees in low-income countries might not have sufficient financial resources to buffer against the shocks created by the COVID-19 pandemic in combination with a drop in oil prices dating to early 2020 [2,3,5]. As revealed in this study, many existing IDPs in Iraq are currently losing their jobs due to extended complications of the COVID-19 pandemic. In particular, information on humanitarian assistance for returnees who have lost jobs due to COVID-19 has become more urgent. As the results of this study suggest, in order to effectively meet the humanitarian needs of displaced households in unexpected situations such as the COVID-19 pandemic, it is necessary for humanitarians and governments to cooperate to identify what humanitarian information they want according to their characteristics [17].

The status of being a female HOH is shown to be a factor in wanting to receive humanitarian information in IDP households. Previous studies have reported that females in the Middle East are more likely to be food-insecure than males. In existing literature, females show lower educational attainment, higher unemployment status, and lower household incomes in comparison to males [18,19]. The proportion of female HOHs in IDP households was higher than the proportion of female HOHs in returnee households in 2020 in Iraq [20]. Female HOHs perceive higher threats than male HOHs to livelihood and are likely to be edged out from competition for employment [3,4,5,6]. Therefore, females are a more vulnerable population in terms of health care needs [9,11]. Most IDPs do not live in camps [12], so some strategies are needed to help female HOHs settle and adapt in camps. A case study in Northern Nigeria on IDPs stressed the supportive communal relationships, seeking alternative means of income, and healthcare services in camp-like settings [13]. Female not having a male HOH who can protect them from threats should be given the opportunity to participate as a member of camps and obtain appropriate health information about themselves and their children within a safe environment [13].

In this study, returnee households with HOHs with health problems were less likely to want information about humanitarian assistance. Despite suffering from physical and/or mental illness, many refugees reported being not able to receive care for their health problems due to long distances from home to health care facilities, lack of adequate health care providers and equipment/medications, and/or long waiting time [2,9,10,11]. Also, in Iraq, private services have been shown to be preferable to refugee camp clinics for primary health care [11]. This might explain why returnees were less likely to want information about humanitarian assistance, such as health care, even in instances of HOHs with health problems. Further research should be conducted among IDPs in camps, IDPs out of camps, and returnees to examine factors associated with decisions to select types of health care in cases of HOHs with health problems.

In this study, returnees who reported being able to walk to the nearest health care facility within 30–59 min had lower humanitarian information requirements than returnees with a walking time of less than 15 min to the nearest health care facility. The distance that people with health problems can walk (which might be no more than 30 min) and the availability of transportation seem to have influenced this finding. Previous studies have shown that IDPs in gathered settings, such as camps, were more likely to receive assistance and protection from humanitarian actors in comparison to refugees in dispersed settings including returnees [21]. Barriers to access to health care facilities reported by refugees include health problems, costs, lack of knowledge about available health care services, and/or inability to get an appointment [9,11]. Also, displaced persons have been shown to have difficulties in accessing health care services due to financial burdens or the absence of key individual documentation [3]. Tailored solutions to provide humanitarian information depending on distance from health care facilities and displacement status will be a challenge, but such solutions have the potential to meaningfully improve the health problems of returnees [21]. In terms of geospatial analyses based on health care facilities, the establishment of temporary, mobile clinics by the Ministry of Health of Iraq and other governmental organizations and non-governmental organizations can help provide humanitarian assistance to returnees [22]. A recent systematic review supported the use of mobile clinics which can provide multiple primary health care services in humanitarian settings [23].

In returnees, greater walking distances from the nearest marketplace were shown to correlate to a greater desire to receive humanitarian information. Most returnees reported needing humanitarian information because of their reduced ability to purchase or access food through markets, resulting in increased nutrition-based health problems [4,5]. Restrictions on movement, disruption of public services, and other measures to contain the spread of COVID-19 have made these issues even more devastating for returnees [3]. Fortunately, overall restrictions on marketplace access have been improved since August 2020 in Iraq [4]. Nevertheless, further research is needed to investigate the extent to which governmental restrictions on the movement of returnees have eased.

The findings of this study will serve as a valuable and comprehensive reference point for international and local policymakers and stakeholders in providing humanitarian assistance. In this study, factors contributing to the desire of displaced people to benefit from humanitarian assistance programs varied among displaced groups. Technology, including online and mobile communications, can be used to provide displaced households with information about jobs, health care services, and transportation to health care facilities or marketplaces by humanitarian actors [5,7]. Most IDP and returnee households have at least one member with access to a smartphone. Accordingly, humanitarian organizations have begun to use mobile technologies and other new technologies to provide humanitarian assistance [24]. As the best example, the Iraq IDPs Information Center offers free, multilingual services for IDPs and refugees to obtain relevant, up-to-date information on a variety of issues [25]. Mobile asset replacement, cash-for-work programs, and legal assistance provided by the government are emerging as priorities to assist displaced persons [3]. Many women have fewer opportunities than men to use technology to receive humanitarian assistance. Married women tend to use the mobile phones of their husbands, and some more conservative women are not willing to provide their own personal information online [24]. Traditional cash and voucher assistance remains a key component of humanitarian assistance for female HOHs to meet their basic needs and access health care services [3].

Regardless, the study has some limitations to interpret. First, the quality of data may differ between face-to-face interview samples and phone-based interview samples. Phone-based interview samples had a relatively high health risk associated with COVID-19 and access restrictions compared to face-to-face interview samples [3]. Interviews using mobile phones have certain challenges with ensuring comprehensive communication and collecting answers to sensitive questions. Therefore, the questionnaire for phone-based interviews should include more specific situations in which humanitarian aid is needed and additional questions about poor health problems. Second, because no distinction was made between displaced households in camps and those out of camps, the differences in characteristics between these two groups are not reflected in the results. Accordingly, caution is needed in generalizing the findings of the analysis. Third, the researcher should be cautious about generalizing the study findings because this study does not adjust the geographical aspects, such as the regional characteristics or areas of origin where displaced people currently live [3]. Finally, some questions related to government services or safety issues might be underestimated or overestimated due to displacement status (i.e., social desirability bias).

## 5. Conclusions

This study investigates factors contributing to whether displaced households want to receive humanitarian information from humanitarian actors during the COVID-19 pandemic in Iraq. Both IDP and returnee households were desirous of receiving information about livelihoods as their top priority. Unemployment in members of returnee households due to COVID-19, being a female HOH, and IDP heads of household with health problems were shown to be urgent issues in Iraq. Under these circumstances, displaced households report needing more humanitarian aid than before. In aspects of humanitarian aid and policy support, financial support (i.e., traditional cash, voucher assistance, job expansion, and other types of financial aid), relief of movement restrictions, transportation support, establishment of temporary clinics, and technology support are needed for displaced populations. The findings of this study are a valuable and inclusive point of reference for international and local policymakers and stakeholders to provide humanitarian assistance for displaced households in Iraq.

## Figures and Tables

**Table 1:** Humanitarian needs, COVID-19 impacts, vulnerabilities, and demographic characteristics of Iraqi displaced households.

Domain		Variable	Response	IDPs (*n* = 2161)		Returnees (*n* = 1513)		Total (*n* = 3674)		Statistic ^†^	*p*-Value
				*n*	Weighted%	*n*	Weighted%	*n*	Weighted%		
Humanitarian needs	Want to receive information from humanitarian actors (top 3 priorities/multiple responses)	Safety and security	No	1570	76.8	1001	64.5	2571	67.0	21.9	<0.001
			Yes	591	23.2	512	35.5	1103	33.0		
		Status of housing	No	1650	84.2	1126	63.4	2776	67.6	72.4	<0.001
			Yes	511	15.9	387	36.6	898	32.4		
		Livelihoods	No	809	35.2	587	31.8	1396	32.5	1.7	0.195
			Yes	1352	64.8	926	68.2	2278	67.5		
		Water services	No	1859	92.2	1194	71.0	3053	75.3	96.1	<0.001
			Yes	302	7.8	319	29.0	621	24.7		
		Electricity services	No	1769	92.9	1037	64.6	2806	70.3	372.7	<0.001
			Yes	392	7.1	476	35.4	868	29.7		
		Education	No	2063	96.3	1413	90.0	3476	91.2	17.2	<0.001
			Yes	98	3.7	100	10.1	198	8.8		
		Health care	No	1426	59.6	1052	78.2	2478	74.4	57.5	<0.001
			Yes	735	40.4	461	21.8	1196	25.6		
		Humanitarian assistance	No	940	36.3	944	68.2	1884	61.8	144.6	<0.001
			Yes	1221	63.7	569	31.8	1790	38.2		
		Legal services	No	2059	93.2	1400	94.3	3459	94.1	0.8	0.386
			Yes	102	6.8	113	5.7	215	5.9		
		House, land, and property services	No	2119	98.6	1469	97.9	3588	98.1	0.8	0.369
			Yes	42	1.5	44	2.1	86	2.0		
	Insufficient food (past 30 days)		No	1874	80.1	1432	97.8	3306	94.2	274.2	<0.001
			Yes	287	19.9	81	2.2	368	5.8		
COVID-19 impacts	Movement restrictions by government	Need to obtain security clearance/coupons	No	2025	93.8	1376	94.2	3401	94.1	0.1	0.788
			Yes	107	6.2	98	5.9	205	5.9		
			Missing	29	0.0	39	0.0	68	0.0		
		Need to show ID documents	No	2046	95.0	1363	89.1	3409	90.3	9.5	0.002
			Yes	83	5.0	109	10.9	192	9.7		
			Missing	32	0.0	41	0.0	73	0.0		
		Time restrictions on when to leave and return	No	1993	89.7	1377	87.2	3370	87.7	1.4	0.242
			Yes	146	10.3	100	12.8	246	12.3		
			Missing	22	0.0	36	0.0	58	0.0		
		Need to provide a specific reason for movement	No	2084	92.1	1409	97.5	3493	96.4	28.6	<0.001
			Yes	53	7.9	69	2.5	122	3.6		
			Missing	24	0.0	35	0.0	59	0.0		
		Physical roadblocks	No	2069	95.9	1390	96.8	3459	96.6	0.9	0.336
			Yes	59	4.1	84	3.2	143	3.4		
			Missing	33	0.0	39	0.0	72	0.0		
	Unemployment of household members due to COVID-19		No	1443	59.8	1192	89.6	2635	83.6	309.8	<0.001
			Yes	705	40.2	292	10.4	997	16.4		
			Missing	13	0.0	29	0.0	42	0.0		
Household demographic/vulnerability covariates	Number of family members *		(min = 1; max = 46)	6.39	(6.15, 6.62)	5.17	(4.98, 5.36)	5.41	(5.26, 5.57)	−8.0	<0.001
	Elderly-headed household (65 or older)		No	1989	92.0	1418	95.0	3407	94.4	5.3	0.022
			Yes	172	8.0	95	5.0	267	5.6		
	Female-headed household		No	1901	87.7	1339	90.3	3240	89.8	2.4	0.120
			Yes	260	12.4	174	9.7	434	10.2		
	HOH with health problems (injury, chronic/communicable disease, etc.)		No	1392	65.1	1005	65.1	2397	65.1	0.0	0.986
			Yes	769	34.9	508	34.9	1277	34.9		
	Employment status of HOH		No	655	31.7	347	18.6	1002	21.2	29.6	<0.001
			Yes	1505	68.3	1165	81.4	2670	78.8		
			Missing	1	0.0	1	0.0	2	0.0		
	Household’s total monthly income (per 100,000 IQD)		(min = 0; max = 70)	4.55	(4.15, 4.95)	5.05	(4.61, 5.49)	4.95	(4.59, 5.31)	1.7	0.100
	Walking time to reach the nearest health care facility		<15 min	1236	59.5	806	54.8	2042	55.7	7.3	<0.001
			15–29 min	632	28.0	447	28.9	1079	28.7		
			30–59 min	177	6.7	116	12.2	293	11.1		
			1–3 h	63	3.5	108	3.8	171	3.7		
			3 h <	53	2.3	36	0.4	89	0.8		
	Walking time to reach the nearest marketplace		<15 min	1800	83.4	1191	75.9	2991	77.4	4.1	0.003
			15–29 min	246	11.7	210	17.6	456	16.4		
			30–59 min	88	3.8	36	4.1	124	4.0		
			1–2 h	20	0.8	25	1.3	45	1.2		
			2 h<	7	0.3	51	1.2	58	1.0		

IDP, internally displaced person; HOH, head of household; IQD, Iraqi dinar. * Weighted mean estimates and 95% confidence intervals are presented. ^†^ Inter-population subgroup comparisons were performed using Rao-Scott corrected chi-square tests for categorical data and sampling design weighted univariable linear regression for numerical data. Strata with single sampling unit centered at overall mean.

**Table 2:** Factors associated with humanitarian information needs among Iraqi displaced households.

Variable	Total					IDPs					Returnees				
	OR	Robust SE	*p*-Value	LL	UL	OR	Robust SE	*p*-Value	LL	UL	OR	Robust SE	*p*-Value	LL	UL
Unemployment of household members due to COVID-19															
No	1.000	ref				1.000	ref				1.000	ref			
Yes	2.061	0.287	<0.001	1.569	2.708	1.307	0.227	0.124	0.929	1.839	2.661	0.526	< 0.001	1.805	3.922
Displacement status															
IDPs	1.000	ref				N/A									
Returnees	0.325	0.040	<0.001	0.255	0.414										
Number of family members															
	0.996	0.024	0.865	0.949	1.045	1.009	0.027	0.749	0.956	1.064	0.988	0.033	0.726	0.926	1.055
Female HOH															
No	1.000	ref				1.000	ref				1.000	ref			
Yes	1.434	0.336	0.124	0.906	2.271	1.971	0.476	0.005	1.228	3.164	1.333	0.432	0.376	0.706	2.516
Elderly HOH															
No	1.000	ref				1.000	ref				1.000	ref			
Yes	1.411	0.445	0.275	0.760	2.620	0.747	0.214	0.310	0.426	1.311	1.833	0.783	0.156	0.793	4.239
HOH with health problems															
No	1.000	ref				1.000	ref				1.000	ref			
Yes	0.759	0.119	0.077	0.558	1.031	0.993	0.180	0.968	0.695	1.418	0.666	0.134	0.044	0.449	0.988
Employment status of HOH															
No	1.000	ref				1.000	ref				1.000	ref			
Yes	0.931	0.181	0.712	0.635	1.364	1.121	0.226	0.571	0.755	1.666	0.887	0.247	0.666	0.514	1.530
Household’s total monthly income (per 100,000 IQD)															
	0.983	0.013	0.198	0.959	1.009	0.953	0.017	0.008	0.920	0.987	0.999	0.017	0.932	0.966	1.033
Walking time to reach the nearest health care facility															
<15 min	1.000	ref				1.000	ref				1.000	ref			
15–29 min	1.315	0.231	0.120	0.931	1.857	1.189	0.224	0.359	0.822	1.720	1.334	0.291	0.187	0.869	2.046
30–59 min	0.454	0.119	0.003	0.272	0.759	1.329	0.410	0.356	0.726	2.433	0.308	0.117	0.002	0.146	0.649
1–3 h	1.370	0.404	0.286	0.768	2.444	1.022	0.394	0.954	0.480	2.176	1.385	0.507	0.374	0.675	2.841
3 h<	0.910	0.461	0.852	0.337	2.455	1.246	0.658	0.678	0.442	3.510	0.839	0.879	0.867	0.107	6.554
Walking time to reach the nearest marketplace															
<15 min	1.000	ref				1.000	ref				1.000	ref			
15–29 min	0.939	0.209	0.776	0.607	1.451	0.634	0.166	0.082	0.380	1.059	1.046	0.277	0.864	0.623	1.758
30–59 min	0.988	0.389	0.975	0.456	2.140	1.072	0.373	0.842	0.542	2.120	1.030	0.516	0.953	0.386	2.752
1–2 h	11.569	9.386	0.003	2.358	56.769	1.217	0.798	0.765	0.336	4.402	17.275	17.106	0.004	2.476	120.500
2 h <	2.789	1.257	0.023	1.153	6.747	0.331	0.342	0.285	0.043	2.519	3.070	1.553	0.027	1.138	8.281

IDP, internally displaced person: HOH, head of household; IQD, Iraqi dinar; OR, odds ratio; SE, standard error; CI, confidence interval; LL, lower limit of 95% CI; UL, upper limit of 95% CI; ref, reference; N/A, not applicable.

## Data Availability

Data was obtained from REACH Initiative and is available with the permission of REACH Initiative.
